# Young Women’s Attitudes and Concerns Regarding Pornography and Their Sexual Experiences: A Qualitative Approach

**DOI:** 10.3390/healthcare11212877

**Published:** 2023-11-01

**Authors:** Mónica Fernández-Ruiz, Olga María López-Entrambasaguas, Jose Manuel Martínez-Linares, José Granero-Molina

**Affiliations:** 1 Nursing, Physiotheraphy and Medicine Department, University of Almería, 04120 Almería, Spain; monica_eme@hotmail.com (M.F.-R.); jgranero@ual.es (J.G.-M.); 2 Nursing Department, Faculty of Health Sciences, University of Jaén, 23071 Jaén, Spain; 3 Nursing Department, Faculty of Health Sciences, University of Granada, 18016 Granada, Spain; jmmartinezl@ugr.es

**Keywords:** pornography, qualitative research, sexual behavior, sex education, sexual health, young women

## Abstract

This study explores female university students’ attitudes toward and concerns about pornography, based on their experience watching it and on sexual encounters with men. It used a qualitative descriptive design. Individual semi-structured interviews were conducted with 22 participants between March and April 2020. Thematic data analysis was performed using COREQ reporting guidelines. Three themes emerged from the data: (1) the sexual learning process, (2) the role of pornography in women’s sexuality, and (3) sexual experiences. Participants reported how they learned about sexuality and how pornography influenced sexual experiences. Self-esteem issues and societal norms regarding hair removal and difficulty saying “no” to unwanted or humiliating sexual practices were found. The young women were not comfortable with women’s representations in mainstream pornography. They blamed pornography for negative sexual experiences and claimed it influenced them and young men. Participants usually assumed submissive roles during sex and permitted aggressive sexual behaviors. The study reveals valuable information on how young women learn about sex and their difficulty in refusing unwanted sexual activities and even aggressive practices. Sexual education programs should include assertiveness training to improve sexual health, consent, and well-being.

## 1. Introduction

Over the past four decades, public worry about the connection between pornography and sexual assault and aggressiveness has spurred debate on a global scale, and this topic continues to be an important and contentious issue today [1]. Mainstream pornography is distinguished from feminist pornography by its emphasis on pleasing a large audience while making a profit [1]. On the Internet, mainstream pornography is easily accessible. Male dominance and aggression and female subservience are common themes in mainstream pornography [2]. De Miguel-Álvarez (p. 380, [3]) calls it “the sex school for the youngest”. According to Owen et al. [4], Internet pornography consumption at very early ages distorts reality in terms of sexuality and sex. Recent studies show that early sexual initiation is common [5]. A recent research review concluded that adolescent sexual and reproductive health is influenced by pornography viewing [6]. Ballester et al. [7] demonstrated pornography’s negative impact on sexual behaviors and gender relationships in young people in Spain. However, a few studies have found that pornography may have a positive effect on adolescent sexual development, such as sexual confidence [8], sexual identification with regard to gender identity and sexual orientation [9], and sexual gratification [10].

Four thousand heterosexual scenes from Pornhub and Xvideos websites were analyzed, and it was found that in 97% of aggressive scenes, the object of aggression was the woman [1], who reacted to these behaviors neutrally or positively in most cases. Moreover, similar conclusions were drawn in a recent systematic review, highlighting that “dominating and violent behaviors were nearly always directed toward women” [11]. This may cause consumers to adopt a sexual script that supports violence against women [1]. Other studies have shown that sexually explicit media (SEM) exposure modifies attitudes toward women and sexuality [12] and is associated with engaging in sexually aggressive behaviors [13]. Healthy sexuality formation is crucial in how we interact with others and ourselves in adulthood. There are aspects of sexuality absent from pornography, such as emotional intimacy, negotiating consent, and discussing contraception; thus, it seems that SEM is not a comprehensive sexuality educator, although presumably, it was not designed or intended to be.

Sexuality is a fundamental human dimension that comprises sexual orientation, sex, gender roles and identity, pleasure, intimacy, eroticism, and reproduction [14]. Sexual health goes well beyond the mere absence of sexually transmitted infections; it includes respect and enjoyment of all sexual rights. Likewise, it influences the physical, psychological, and emotional well-being of people [14]. The development of desire, sexual motivations, and affective and sexual bonds are influenced by the environment and context surrounding young people [15]. Farré et al. [5] reported that adolescents’ psychosexual development may be affected by pornography exposure.

More than 50% of Spanish adolescents aged between 14 and 17 are regular pornography consumers [16]. In a recent Spanish investigation [17], it was reported that boys are the main pornography consumers (86.9%) compared to girls (54.8%), with a consumption initiation age of 8 years. Also, pornography consumption’s impact on interpersonal relations is greater for boys than for girls. Other Spanish authors [18] highlighted the need for a high-quality and comprehensive sex education, as there are still myths and misinformation about sexuality, and the current sex education programs continue to focus only on unwanted pregnancy and sexually transmitted infection (STI) prevention. Finally, García-Vázquez et al. [19] gathered data on the evolution of sexual health in the Spanish adolescent population, which included the use of contraceptives, the number of sexual partners, coition and first sexual intercourse, voluntary termination of pregnancy, STIs, and the number of victims of gender violence. Few studies have been conducted on pornography and sex education in Spain.

There are only a few qualitative studies on women’s perception or experiences regarding pornography. The pornography-related narratives are diverse and reveal both positive and negative aspects of its consumption. When considering solitary versus partnered sexuality, the opinions on pornography diverge. Of the small subset, two Australian studies recently explored women’s experiences and pornography’s interaction with intimacy, relationships [20], and pleasure [21]. Additionally, women’s orientation toward pornography was outlined in a Canadian study [22] and in another Australian quantitative investigation [23]. However, to date, we have found no qualitative studies that explore issues related to women’s emotions, experiences, perceptions, and attitudes toward sexuality and, specifically, pornography, in Spain. This is increasingly important, because in Spain, pornography has been recently defined as a sex educator [3]. The present research addresses the gap in regards to women’s experiences, perceptions, and attitudes towards sexuality and pornography, exploring the gendered dimension of mainstream pornography.

## 2. Materials and Methods

### 2.1. Design

A qualitative research approach was used in this study. The exploratory and descriptive nature of the study allowed us to address people’s realities and understand their perspectives. Qualitative research seeks to comprehend, describe and, at times, explain social phenomena from the perspective of the individual as well as society as a whole [24], recognizing the interpretive nature of data coding [25]. Qualitative work may be useful in explaining behaviors and attitudes toward sexuality.

### 2.2. Participants

The participants were women and were required to (1) have had sexual relationships with men, (2) have watched pornography, and (3) be studying at the university or have some level of university education. The third inclusion criterion was based on convenience, as the main researcher was studying at the university and was well acquainted with the social setting there.

Participants were selected through the non-probabilistic method of snowball sampling and were first contacted via phone calls. The sampling procedure began with one woman who provided five referrals. Then, each new referral provided the main researcher with more potential referrals. The chain sampling sequence was repeated until the study had a sufficient number of participants (until data saturation was reached). Participants’ ages ranged between 19 and 29 years, and they were all Spanish. Twenty-two young women participated. The participants’ sociodemographic characteristics are shown in Table 1. None dropped out of the study.

### 2.3. Data Collection

Data were collected online between March and April 2021. The instrument used to collect data was semi-structured individual interviews. The main researcher adapted herself to the electronic resources that the participants had; thus, the interviews were conducted via Skype (version 8.67.0.99), WhatsApp Messenger (version 2.19.118 (452771)), Facebook (version 303.0.0.30.122 (269803905)), Discord (version 26.1), and Google Duo (version 87). The researcher ensured the establishment of continuous contact, listened carefully, and showed interest in what participants said.

The question script was designed ad hoc based on a literature review, and two content blocks were distinguished. Eight questions addressed participants’ sexual experiences and sex education. Another eight questions specifically related to pornography. Part of the script is shown in Table 2.

Participants received a consent form via email, and they also had the option of clarifying any doubts before starting the interview. Twenty-two interviews, with an average duration of 45 min, were conducted. Data collection was terminated when no additional topics were identified and data began to repeat, signifying that an adequate sample size was reached (data saturation) [26]. The interviews were audio-recorded and transcribed verbatim. Participants were given the chance to check the content of their transcriptions and the recrafted stories derived from them. This way, participants could validate their contributions and the meaning of their experiences. They all confirmed that the stories were documented in an appropriate manner.

### 2.4. Data Analysis

Braun et al. [27] offered three typologies of thematic analysis (TA), as it is considered a theoretically flexible method. The reflexive approach to TA was used in order to emphasize the active role of the researcher in knowledge production [28] and the predominance of the inductive approach.

Reflexive thematic analysis was performed to analyze data, and it includes six steps [28,29]: (1) transcription, reading, and re-reading (familiarization); (2) initial generation of codes (61 codes were initially generated); (3) conversion of codes to themes (by discarding the codes that were not significant, 61 codes were reduced to 45); (4) revision of themes and generation of the conceptual analysis map; (5) labeling of the themes and determining the analysis structure (three themes and six subthemes); and (6) elaboration of an analysis report. ATLAS.ti software (version 8.0) was used to facilitate the analysis.

Investigator triangulation was developed to provide multiple observations and different perspectives and to gain a comprehensive understanding of phenomena [30]. The initial coding was performed independently by the first and the second researchers. To describe and understand all aspects of the content, they made notes and added headings within the texts. The codes were subsequently agreed upon with a third researcher (J.G.M.), after which the final number of codes was reached. The initial generation of themes was also conducted by two researchers, and the final tree of subthemes and themes was agreed upon with the third researcher. Thus, the three researchers compared the initial codes and their interpretations of the results, and emerging themes were discussed until they reached consensus. No modifications were required after participants verified the transcriptions’ content.

### 2.5. Ethical Considerations

Informed consent was obtained from all participants before commencing interviews. The ethical standards established by the Declaration of Helsinki were followed, and personal data were processed following Regulation (EU) 2016/679 of the European Parliament and of the Council of 27 April 2016, on the protection of natural persons concerning the processing of personal data and the free movement of such data, and repealing Directive 95/46/EC. This study received approval by the Ethics and Research Committee of the Department of Nursing, Physiotherapy and Medicine (No. 120/2021).

## 3. Results

The testimonies of 22 women with some level of a university education from different scientific fields were analyzed. Most were heterosexual (86.36%). The analysis resulted in the emergence of three themes and six subthemes (see Table 3).

### 3.1. Theme 1: The Sexual Learning Process

The following quotes describe the process through which participants learned about sexuality from early childhood to the present time. In addition to discussing themselves, they offered their thoughts on the most common forms of sexual learning.

#### 3.1.1. Subtheme 1: Educational Agents

The educational agents were neither the school nor the family, with a generalized consensus on this aspect:

*“I could not talk about sex with my parents, that was inappropriate and taboo”.*

(I22)

*“My father once told me, ‘Shut up, girl, we don’t talk about that’”.*

[I13]

Only one participant commented on a female teacher from whom she had learned a lot. According to this participant, the teacher was “very alternative and spoke a lot about sexism, homophobia…” (I8). Most stated that their sexual learning occurred mainly in their group of friends and in their experiences with partners; the latter grew over the years, after having a diversity of sexual encounters:

*“I like to talk with friends [about sex] because I can learn from their experiences. For example I like to know what practices give them pleasure, so I can try them and get the experience to know if I like it or not”.*

(I12)

*“Ninety percent of what I know […] I learned it with my boyfriend. In fact, I did not even enjoy this. Before that, sex was something I heard [about], but that was it”.*

(I5)

*“My first boyfriend was who taught me about sex, as he already had experience, so the sexual information I first got was from him”.*

(I1)

Another way to obtain information and knowledge was through books, articles, or pornography:

*“Reading articles, Instagram accounts, I’ve watched porno […]”.*

[I7]

*“There was a time when, rather than watching pornographic videos, I preferred reading erotic novels and using my imagination”.*

[I2]

*“For me, pornography was the most direct way to understand what sex was all about”.*

[I13]

#### 3.1.2. Subtheme 2: Obstacles and Facilitators on the Internet

Some participants also stated that having a partner or having free access to the Internet and social networks can sometimes impede healthy sexuality and lead to sexual miseducation. A number of participants thought that sexual content on the Internet is coitus-centered and partnered and that there is a need to broaden the focus beyond an intercourse-centric perspective and include a variety of sexual behaviors:

*“The only thing that appeared to be important at first was intercourse, when I was younger I was obsessed with it, with getting it right, you know? But as you get to have sexual experiences with more people, you discover new things”.*

(I3)

*“My first experiences with intercourse were disappointing, because I expected it to be extremely pleasurable because I had seen women enjoying penetration on the Internet! Adolescents should have access to more holistic sexual education and be aware of the realities of sexual relationships and how diverse they can be”.*

(I4)

Some women specifically referred to the negative consequences deriving from the absence of proper sex education during childhood/adolescence and the scarce parental control in terms of surfing the Internet:

*“Because there is no sex education and young people need to know… so, at the age of 13 or 14 years, they search for it [sexual information], and I believe that it is very normal that they end up watching porno”.*

(I12)

*“There are a lot of videos on the Internet that are not appropriate for a child to watch, like, for example, physical aggressions, sex with animals, or violations. I don’t know how it will affect people, but that can’t be good for their psychological development”.*

(I18)

However, there were participants who thought that the Internet could improve the sexual health literacy of the population. Furthermore, some Instagram accounts had assisted them in improving their sexual knowledge:

*“Thanks to social networks, everyone has come out to give speeches and share their experiences. Before that, it was more of a taboo; nobody talked about it, and it seemed to be inappropriate. Ever since the feminist liberation, such taboo is disappearing”.*

(I6)

*“Instagram accounts such as Platanomelon, Diversual, and Mama Casquet, for example, promote healthy sexuality and provide a lot of information, I love them”.*

[I7]

### 3.2. Theme 2: The Role of Pornography in Women’s Sexuality

The process through which participants learned about sexuality from early childhood to the present time was explored. The sexual experiences of participants were largely influenced by pornography, either by their use of pornography or by their sexual partners. They all had similar opinions about the content of pornography. They did not reject it but demanded urgent changes.

#### 3.2.1. Subtheme 1: Use of Pornography

Participants were asked about their pornography consumption. The reasons for this are diverse: quick masturbation due to a lack of imagination, curiosity, to become aroused, or to learn about sex. Thus, they revealed the educational role of pornography:

*“At the beginning, I watched it a little bit, just to learn, and then I watched it to masturbate”.*

(I7)

*“I began watching it out of curiosity, because it was such an unknown world and it seemed to be the most realistic thing I could see to learn”.*

[I20]

*“I needed a stimulus to get excited, because if I use my imagination, I get distracted and lose the arousal”.*

[I10]

Many participants spoke in the past tense, highlighting that they currently consumed it very rarely since the content did not appeal them. It was as if pornography served a purpose at one time but was no longer relevant to them today:

*“The truth is that I have rarely looked for a pornographic film; I don’t know if it’s been at least 6 or 7 years”.*

[I17]

*“I don’t find it very appealing; I almost feel as though I don’t need it anymore”.*

[I19]

Another reason to consume pornography as pointed out by the participants was, in rare cases, when their partners proposed it in a sexual encounter:

*“When I watched it [pornography], I was with someone who proposed it [the partner]”.*

(I1)

#### 3.2.2. Subtheme 2: Conception of Pornography

We asked, “What audience do you consider current mainstream pornography to be aimed at?”. Without reservation, the participants answered that it is aimed at men and that the role of the woman is that of subordination and reification. They disliked how women are treated in many cases, and they also mentioned unrealistic practices:

*“It’s sexist and outdated, and it brings the woman down to a very inferior position, where she is only an object”.*

[I2]

One bisexual participant expressed anger when stated that lesbian pornography is not aimed at homosexual women:

*“Lesbian sex is not for lesbian women; it is made for guys, because it is a fantasy of theirs that two women have sex with each other, and then they feel they are entitled to ask a lesbian couple to do a threesome with them”.*

[I10]

Some participants whose work was related to social awareness regarding prostitution thought that current pornography promotes the seeking of prostitution services by young men, which they felt is upsetting. These opinions were based on their work experiences with prostitutes:

*“They [young men] want to perform a sexual act like the ones they see in porno, but they can’t do that with normal women [women who are not prostitutes], because they see it [as] inappropriate or weird, so they go with prostitutes. They pay them, and then they do whatever they want”.*

[I6]

*“Pornography consumption is increasing among young guys, the same as prostitution consumption. After binge drinking in the street, they go in search of the woman”.*

[I9]

Most participants considered pornography to distort what real sexuality was or how it should be. Almost all of them described, without providing much detail, some personal experiences in which the man attempted to carry out a fictional sexual practice:

*“It really bothered me a lot when some guys tried certain things because they’re like ‘uff’ [exclamation that denotes intense desire] I’m not [in] a porno video, dude! We are a man and a woman having sex; that’s it”.*

[I17]

*“A guy once asked me if I minded if he ejaculated on my face, what the hell?! No man, thanks for asking, don’t you dare”.*

[I13]

Some participants also mentioned the great contradiction between what is promoted in mainstream pornography and the current strength of the feminist movement. The messages generated from these two movements were valued as opposites, with young people receiving both:

*“I believe that porn will have a negative impact on society. It’s a bit contradictory because we’re trying to educate our society, in the best way we can, as a feminist society, but pornography does not promote male–female equality”.*

[I4]

The majority of remarks made about pornography were negative; however, when participants were asked to explain and argue about whether they supported or rejected pornography, none rejected it outright. It taught them about sex in some ways, and they enjoyed it as well. What they wanted was more control over the age of access and the removal of certain content (such as rape, the submissive role of women, pederasty, and zoophilia). Their comments highlight how they perceived the potential educational role of this sexually explicit content:

*“People have mobile phones at very early ages, without any knowledge about sexuality, so they are freely watching loads of videos that may not be appropriate”.*

[I14]

*“There are very nasty things, even women being beaten up and things like that, which I do not think should be on the Internet […] nowadays children have easy access to the Internet”.*

[I15]

However, so-called feminist pornography is highlighted as positive and less harmful with regard to the sexuality of those who consume it. This type of pornography is closer to participants’ tastes and more realistic in terms of what they consider sexual relations. Another positive aspect of feminist pornography is that women do not suffer and are not defiled in any way:

*“It’s porn for everyone; for women, men, transsexuals, and people of any sexual orientation, where everyone feels pleasure, however it is not easy to find free videos on the Internet”.*

[I22]

*“I’ve seen feminist pornography videos, and I believe they depict a more realistic scenario of what constitutes a sexual relation”.*

[I10]

### 3.3. Theme 3: Sexual Experiences

This theme addresses participants’ conceptions of how complete and high-quality sexual intercourse is or should be and how they experience their sexuality and sexual encounters, as influenced by pornography or compelled by current society.

#### 3.3.1. Subtheme 1: The Conception of Sexual Encounters

Participants presented very similar views on how complete, healthy sexual intercourse should be. Experimentation, time passing, and experience in different situations were the main sources of education. Interestingly, many had to go through unpleasant sexual encounters to understand how a healthy relationship functions. They began having sexual relations focusing on coitus, although with time and experience, they considered that, even without coitus, all sexual practices are sex:

*“What one sees at the beginning is simply the coitus, because that’s what really matters, but obviously, in time and after meeting different people, one discovers other things”.*

[I1]

*“Ninety percent of what I know […] I learned it with my boyfriend, my first stable relationship. In fact, I did not even enjoy sex before that”.*

[I5]

All participants related satisfaction to orgasm; thus, sexual intercourse without orgasm would not be fully satisfactory:

*“For me, it’s important because it’s the peak of pleasure. If I don’t finish, it’s like something is missing”.*

[I4]

They also highlighted that it is important for both partners in the couple to obtain pleasure during sexual intercourse and to build trust and communication in order to behave naturally and experience enjoyment:

*“The first time I was with someone [having sex] I did not enjoy it at all, but in time, I know myself better and I’m comfortable, so everything flows and I don’t have to think or worry about anything”.*

[I3]

*“I had to say to him ‘hey, are we having sex or are we doing porn?’ We are two people having sex, this is not only about you”.*

[I16]

#### 3.3.2. Subtheme 2: Emotional Experiences

Lastly, we present the results relative to participants’ experiences in their sexual encounters from the lens of the most intimate and emotional feelings as well as how they acted in certain situations. These situations involved medium or high degrees of discomfort, especially in casual sexual encounters, where they accepted male behaviors toward them that they disliked. This subtheme is subdivided into three sections: self-esteem, socially imposed hair removal, and difficulty saying no.

##### Self-Esteem

All participants interviewed in the present study reported that they had physical complexes that prevented them, at some point, from having a pleasant sexual relationship. Some of them were not comfortable with the appearance of their genitals or with their body size. Fear of rejection, the need to switch off the light, and refusal to assume certain positions are reflected in the following comment:

*“I tried to meet guys in the night. I was ashamed of my body and feared that they thought ‘wow, this woman is fat’… and I have been more worried about me than about enjoying that moment, and there are positions… Actually, I have felt bad, sometimes uncomfortable”.*

[I6]

##### Socially Imposed Hair Removal

Another aspect emerging during the interviews was hair removal. The participants expressed that they did not care whether their partners had shaved/waxed; however, in many cases, they felt that being shaved/waxed was a requirement to have sexual intercourse with a man:

*“I have been in situations in which (guys) told me ‘if you’re coming with me you have to come prepared’, meaning ‘if you come with hair, forget about it’”.*

[I1]

They stated that this social imposition was stronger in younger males, and they blamed this on the influence of early pornography consumption:

*“When my sister was 13 years old, she had a boyfriend who asked her if she was shaved and asked her to send him pictures of her pussy; he told her that if she didn’t do it, his friends would laugh at him”.*

[I2]

The participants knew that hair removal was a social imposition; however, they said they did it because they liked it, arguing that it was more hygienic or that it must be done in accordance with the sex videos circulating on the Internet. An extreme case is that of a participant who experienced health problems related to a preoccupation with intimate hygiene:

*“If I went to bed with a guy, I trimmed it because I thought that was what had to be done, and I also asked him to do it. I washed very frequently with soaps and all that, and I ended up with many infections”.*

[I16]

##### Difficulty Saying “No”

All participants spoke of some sexual experience in which they performed a practice that they disliked. This practice was initiated by the man, with no previous warning or negotiation, which made participants feel uncomfortable, upset, or even humiliated. It is surprising that only one participant, on one occasion, told the guy to stop because she did not like what he was doing. The rest of the participants expressed difficulties stopping a sexual act that made them feel uncomfortable or bad:

*“I didn’t want to say no to him and I couldn’t say no, but I wanted him to finish because I felt horrible; I didn’t want to be there, I wanted to leave […] even in a stable relationship I‘ve also felt that I didn’t want to do it, but I did it because he wanted to”.*

[I11]

Some participants, while talking about their experiences, realized that their behavior was similar to women in pornographic videos, because they had passive behavior and accepted any practice. Some reasons stated by the participants to justify this difficulty in saying included considering the uncomfortable practice as “normal”. Therefore, they said nothing and uncomplainingly accepted that “that is part of sex”, as if they were obliged to do something they dislike:

*“Maybe I’ve felt uncomfortable sometimes… in some cases, I’ve done something in sexual intercourse because it’s normal, everybody does it; and so, if everybody does it, even if I didn’t like it, I ended up doing it, although it never convinced me”.*

[I21]

Another reason stated was to please a partner who insisted on having sex, although the women did not want to. The final result was that they felt bad for attending to the needs of the other instead of their own needs:

*“I wasn’t really into it; I didn’t really want to do it. However, for the other person I said yes, because he insisted a lot, but after we did it, I felt bad, thinking that I should not have done it”.*

[I17]

Other participants admitted that they had agreed to a sexual act that they disliked or did not stop their sexual partner for fear of being judged and rejected:

*“[About anal sex] I didn’t like it and I wanted him to stop, but I felt that if I told him ‘hey, stop’ or ‘I don’t want to do it’ he would think I wasn’t good at sex or that I was a prude”.*

[I16]

They also shared some casual sexual experiences in which they felt vexed:

*“Once, a boy held my head and he didn’t tell me he was coming; it was horrible. He didn’t tell me before ‘I’ll let you know and you step aside’ or anything else; he didn’t say anything and I felt very bad”.*

[I19]

Additionally, aggressive sexual experiences, which are frequent in pornography, were not welcomed by the participants:

*“For me, it didn’t make any sense, because there was no affection at all. It was rough and feelingless, with no kisses. I don’t like that. It was too aggressive. I even bled”.*

[I5]

## 4. Discussion

This study aimed to explore female university students’ attitudes and concerns toward pornography, based on their experience watching it and on sexual encounters with men. From the analysis of the results, we obtained information about perceptions toward pornography as well as the participants’ own sexual experiences and behaviors, which were gathered into three themes: “the sexual learning process”, “the role of pornography in women’s sexuality”, and “sexual experiences”.

When the participants in the present study wanted to satisfy the sexual curiosities that emerged during adolescence, that is, when partner selection and exploring their social and sexual identities surged [31], the Internet and pornography became sources of “sex education” [3], exerting a direct influence on them and their sexuality [32]. According to participants, experimentation, time passing, and experience in different situations were the main sources of valuable sex education. Interestingly, many of them had to go through unpleasant sexual encounters to understand how a healthy relationship functioned. Moreover, they pointed out the double-sidedness of free Internet access: on the one hand, it acts as a facilitator of knowledge and the feminist movement, and on the other hand, it is a barrier to a comprehensive sexual education due to mainstream pornography [33].

De Miguel [3] highlighted that mainstream pornography teaches men and women different things. Men are taught what a woman is and what she is for, whereas women are taught the hegemonic physical model they must adopt and that their role is reduced to pleasing the man at any price. Our participants, without a doubt, conceptualized pornography as undermining a healthy sexual relationship. Furthermore, their most intimate experiences and feelings referred to insecurities about their bodies [34] and problems being assertive with men and refusing practices they disliked. Johansen et al. [35], in their study with Danish women, found a concern among participants about being judged negatively if they did not show a liberal approach to sex; they worried about being called “boring” or “prudish”.

Pornography consumers may develop unrealistic sexual beliefs and values [4]. Our participants suggested that those who consume pornography demonstrate an approach to women during sexual encounters that attempts to imitate what they see in pornographic videos [35], which the women dislike. Therefore, it appears that the participants’ negative attitudes towards pornography have been formed through sexual experiences with men, whom they believe are pornographers, and this has resulted in unsatisfactory sexual encounters. It is interesting to see in their testimonies how, on the one hand, they criticize pornography and believe it has a negative impact on women because it influences the sexual behavior of men, and on the other hand, they do not entirely reject it because it has, in some way, served to excite them and educate them about sex practices. At the same time, participants expressed concern about the sexual education of children and adolescents, believing that access to pornography occurs at younger ages and being aware of the long-term harm it may cause.

Dwulit and Rzymski [36] found more pornography users among girls than boys. However, other authors have highlighted that men consume more pornography than women [4,17]. These results were in line with our results, since all the participants had watched pornography, although none considered themselves regular consumers. However, they did value pornography for women more positively. Women react more positively to non-sexist and/or explicitly feminist pornography [37], as well as to pornography that focuses on women’s pleasure or enjoyment [38]. As in previous studies, our participants showed negative affective responses to the types of pornography that they found problematic [39]. In any case, they had a clear opinion about certain visual elements that exist on most free pornography websites: the videos that violate human rights and people’s sexual rights should be removed. As argued by Tarzia and Tyler (p. 3, [40]), “pornography facilitates, exacerbates, or perhaps even causes men’s sexual violence against women”.

Women’s experiences of intimate partner sexual violence “and their sense of direct connection to pornography consumption” is an underexplored area [40] (p. 6), and our participants shed some light on this gap. When they discussed their negative sexual experiences, they did not judge or criticize the man or the sexual act, and they never used the word “violence”. However, they did describe what they intimately felt (vexation, humiliation, being treated as a sexual object) and, in agreement with the aforementioned authors, they associated men’s pornography consumption with these sexual attitudes, of which they disapproved. They justified the men’s actions in most cases, thinking that what they do must “be normal” (or “it is what is expected of me as a woman”). In their testimonies, two important aspects appeared: (1) the difficulty in refusing to perform sexual practices that they dislike or stopping an unwanted action toward them and (2) fear of rejection, which paralyzes them and prevents them from respecting their own wishes. We could not accurately determine whether the difficulty in saying “no” is related to the fact that women assume and internalize the submissive role played by women in pornography, or whether it is due to a deficit of social skills or other factors. However, the Parliamentary Assembly of the Council of Europe adopted a Resolution stating that pornography undermines women’s self-determination and gender equality “by conveying an image of women as subordinate to men and as objects, and trivialising violence against women” [41]. Lundgren and Amin [42] suggest that low consent-negotiation skills and a lack of positive communication relate to dating violence. Moreover, the results of Johansen et al. [35] align with ours, as they found that women were concerned about being treated with respect and their needs being considered in sexual encounters. A positive association between pornography use and sexually objectifying behaviors has been reported [43]. Adolescent men tend to report being perpetrators of sexual coercion more frequently, even be willing to commit sexual violence against their partner [44]. Apostolou and Khalil [45] also found a much greater desire to engage in aggressive or humiliating sexual play in men than in women. The most recent evidence we found regarding the impact of pornography on women/girls is a study commissioned by the European Parliament’s Policy Department for Citizens’ Rights and Constitutional Affairs, the main findings of which include girls reporting that easy access to violent pornography is influencing boys’ sex understandings and expectations [46].

In participants’ testimonies, there arose a novel issue: pornography’s influence on hair removal. A study conducted in New Zealand [47] revealed factors of hair removal practices among men and women, and the authors found no relationship between pubic hair removal (PHR) and regular viewing (or reading) of pornography. However, societal norms (hairlessness and femininity) were the most commonly identified reasons for women’s hair removal in our study. Moreover, Stone et al. [48] reveal partners’ preferences and partners’ consumption of pornography as reasons for women’s PHR. Along these lines, our results showed that societal and gender norms were the main reasons for women’s PHR, specifically and very significantly, the partners’ preferences (even constituting an indispensable requirement) and hygiene. Regarding the fashion of hair removal practices in Western society, Williamson [49] highlighted the potential negative consequences of PHR on health.

In Spain, there is a considerable lack of effective sex education and dating violence prevention programs, as stated by the participants, even though sex education has been a cross-curricular subject for decades. Teachers in schools and high schools claim to have very little training in the scope of sex education [50]. With respect to nursing, there are very few Spanish studies that explore the knowledge and competency level of these professionals in terms of effective sexual education. We found only one study, in which participants demanded more training [51]. Therefore, it is fundamental to understand the sexuality of young people, as well as their practices and needs, to adjust health education programs aimed at sexuality.

### Limitations

Ethnicity and religion are factors to consider in participant selection, as they could have influenced our results. Furthermore, it should be highlighted that the researchers’ first idea was to perform focal groups, in addition to individual interviews. Focus groups would likely generate more interesting results. Participants in focus groups are encouraged to share their opinions, explain why they have them, and disagree with others. As a result, perspectives and experiences that may not be expressed in individual interviews are shared. Moreover, due to the pandemic, the principal investigator was forced to gather the information virtually instead of face-to-face; this may have altered some participant answers, as face-to-face interviews foster a stronger sense of closeness and trust among people. Finally, it should be noted that the findings of this study are not applicable to the rest of the population.

Future research on the phenomenon of sexual violence, sexual coercion, the inability to say “no”, and the societal norms and expectations imposed on women could clarify the study object. Additionally, qualitative research on young men would clarify the many aspects suggested in this study.

## 5. Conclusions

Young Spanish women are not comfortable with the role women represent in mainstream pornography. They reject sexual violence against women and the submissive role they play. Nevertheless, they subconsciously adopt socially expected/pornographically normalized behaviors, such as hair removal or submission to the male partner’s sexual desires, due to the fear of rejection and judgment. Thus, the participants provided clear insights into the aspects of sex education that must be considered. On the other hand, they are not categorically opposed to pornography, as it has helped them learn about sex and, at times, served as a source of pleasure. In fact, some women defend feminist pornography because they believe it provides a more realistic representation of sexual relationships and feminist values. Professionals could apply the study results to the public/private system of sex education, including education on gender-based violence, making responsible choices regarding communication around sexual behaviors, and rights-based approaches to sex.

## Figures and Tables

**Table 1:** Sociodemographic characteristics of the participants.

Participants	Age	Marital Status	Sexual Orientation	Studies
I1	25	With a partner	Heterosexual	Master’s Degree Program in Teacher Training in Spanish as a Foreign Language
I2	25	Single	Bisexual	Early Childhood Education
I3	27	With a partner	Heterosexual	Physiotherapy
I4	25	With a partner	Heterosexual	Criminology
I5	24	With a partner	Heterosexual	Master’s Degree Program in Psychopedagogy
I6	25	With a partner	Heterosexual	Psychology
I7	23	Single	Heterosexual	Bilingual Primary Education
I8	22	Single	Heterosexual	Social Education
I9	25	With a partner	Heterosexual	Social Education
I10	23	Single	Bisexual	Master’s Degree Program in Sexology Sciences
I11	19	With a partner	Heterosexual	Degree of Law
I12	19	Single	Heterosexual	Early Childhood Education
I13	26	With a partner	Heterosexual	Early Childhood Education
I14	26	With a partner	Heterosexual	Master’s Degree Program in Occupational Risk Prevention
I15	21	Single	Bisexual	Nursing
I16	26	With a partner	Heterosexual	Early Childhood Education
I17	29	Single	Heterosexual	Early Childhood Education
I18	25	With a partner	Heterosexual	Pharmacy
I19	26	With a partner	Heterosexual	Criminology
I20	25	With a partner	Heterosexual	PhD Program in Chemistry
I21	27	Single	Heterosexual	Master’s Degree Program in Early Intervention
I22	26	With a partner	Heterosexual	Law

Source: Prepared by the authors.

**Table 2:** Questions of the interview script.

Content	Questions
Sex education	How did you learn what you currently know about sexuality?
Pornography	Do you consider that pornography influences the sexual behavior of those who consume it and that of their partners?
Pornography	What is your opinion on pornography?
Sexual experience	Could you tell me about a negative sexual experience or a sexual encounter in which you did not feel comfortable?

Source: Prepared by the authors.

**Table 3:** Codes, subthemes, and themes.

Codes	Subthemes	Themes
Family, friends, educational center, pornography, prevention speeches, experience with a partner, sex education, Internet, feminist movement, teachers, social networks	Educational agents	The sexual learning process
Social networks, current pornography, easy access to the Internet, slow learning, self-learning, non-existent parental control, taboo, wrong beliefs, experience with a partner	Obstacles and facilitators on the Internet	
Unidirectional pleasure, male pleasure, male dominance, stereotyped roles, female subordination, wrong learning, sexual abuse, female reification feminist pornography, rejection, eradicate, change, acceptance	Conception of pornography	The role of pornography in women’s sexuality
Consumption, arousal, masturbation, lack of imagination, learning	Use of pornography	
Foreplay, confidence, innovating, mutual pleasure, sexual practices, sexual encounters, sexual consent	Conception of sexual encounter	Sexual experiences
Socially imposed hair removal (genital and body hair removal, obligation to wax, requirement to have sex, hygiene). Self-esteem: body rejection. Not saying no: practices with pain, aggression, humiliation, vexation, discomfort, sexual objectification, guilt, normalizing the situation, pressure, non-existing pleasure, coitocentrism, termination of the sexual relation, not verbalizing, unidirectional relations.	Emotional experiences	

Source: Prepared by the authors.

## Data Availability

The data presented in this study are available on request from the corresponding author. The data are not publicly available due to ethical reasons.
